# Association between HER2 expression, genomic characteristics, and tumor immune microenvironment dynamics in epithelial ovarian cancer

**DOI:** 10.3389/fonc.2026.1867768

**Published:** 2026-07-20

**Authors:** Julia Salinaro, Payton De La Cruz, Shriya Perati, Julia McAdams, Samantha Buyungo, Janina Pearce, Areta Bojko, Angelica Salaverria, Kamaljeet Singh, Paul DiSilvestro, Cara Mathews, Nicole E. James

**Affiliations:** 1 Program in Women’s Oncology, Women and Infants Hospital, Providence, RI, United States; 2 Department of Obstetrics and Gynecology, Warren-Alpert Medical School of Brown University, Providence, RI, United States; 3 Therapeutic Sciences Graduate Program, Brown University, Providence, RI, United States; 4 Department of Pathology, Women and Infants Hospital, Providence, RI, United States

**Keywords:** epithelial ovarian cancer, HER2, PD-L1, trastuzumab deruxtecan, VEGF

## Abstract

**Background:**

Despite the rapid clinical approval of the HER2-directed antibody-drug conjugate trastuzumab deruxtecan (T-DXd) for patients with HER2-expressing epithelial ovarian cancer (EOC), preclinical mechanistic studies are lacking.The goal of this investigation was to determine the genomic and immunogenic characteristics associated with HER2 expressing EOC in order to better understand the mechanism of treatment response and what subset of patients will benefit most from single agent and combinatorial HER2-directed treatment regimens.

**Methods:**

130 EOC patients were retrospectively identified from our institution’s internal clinical genomic database. Selected genomic characteristics were stratified by gastric HER2 score and distributions were analyzed by Fisher’s exact test. A subset of 34 EOC tumors were internally HER2 stained and fluorescent immunohistochemistry analysis of PD-L1, CD4, and CD8 was performed. EOC cell lines were treated with T-DXd and *PD-L1* and *VEGFA* levels were assessed via quantitative PCR. VEGF expression following combinatorial T-DXd and bevacizumab treatment was determined via western blot.

**Results:**

Non-significant differences were detected in HRD, *CCNE1* amplification, and *ARID1A* status between HER2 high (n=29) and low (n=101) tumors in an EOC and high grade serous ovarian cancer (HGSOC) sub-cohort (n=98). Although not statistically significant, patients with HER2 high tumors had lower levels of FOLR1 positivity and higher levels of PD-L1 positivity in both EOC and HGSOC cohorts. Intratumoral PD-L1 expression and CD4+ T cell levels were significantly higher (p<0.05) in HER2 high tumors. Finally, T-DXd substantially downregulated *PD-L1* and *VEGFA* expression, and bevacizumab and T-DXd synergistically reduced VEGF expression.

**Conclusions:**

HER2 high EOC tumors are more likely to be FOLR1 negative and PD-L1 positive, and treatment with T-DXd downregulates *PDL-1 and VEGFA* expression. These findings support further examination to determine if immunotherapy and anti-angiogenic treatments synergize with T-DXd in EOC.

## Introduction

Epithelial ovarian cancer (EOC) is a lethal malignancy with a five-year survival rate of only 52% in 2026 (1). While the majority of patients with EOC initially respond to platinum-taxane chemotherapy, most will experience a chemoresistant recurrence 12–18 months following the initiation of frontline treatment (2). Despite the introduction of anti-angiogenic therapies and poly (ADP-ribose) polymerase (PARP) inhibitors in the maintenance setting, there have been minimal improvements in the five-year survival for EOC for the vast majority of patients (3). Therefore, developing more effective treatments in the recurrent setting remains an unmet need.

In recent years, the development of antibody-drug conjugates (ADCs) has emerged as a promising treatment approach to combat therapy resistance for patients with EOC. Currently, there are over 600 ADCs in various phases of clinical development globally (4). While the rapid and ever-evolving clinical development of this novel drug class has garnered excitement, collectively there has been a general lack of preclinical mechanistic and translational endpoint evidence to accompany growing clinical data. A clinically timely example within the field of gynecologic oncology is the DESTINY-PanTumor02 Phase II trial that investigated trastuzumab deruxtecan (T-DXd), an ADC that targets human epidermal growth factor receptor 2 (HER2). The response rate was 45% for patients with HER2-expressing EOC (5). This is in contrast to prior studies of HER2-directed therapies in EOC which demonstrated response rates of less than 10% (6, 7).

While the results of DESTINY-PanTumor02 were encouraging and led to a pan-cancer approval of T-DXd for HER2 expressing tumors, including EOC, it remains unclear as to why patients with EOC responded to T-DXd but not to other HER2-targeting agents. Specifically, how HER2 scores are associated with other somatic mutations and tumor microenvironment characteristics in EOC has not yet been characterized, making it difficult to address this question. Furthermore, without compelling preclinical data, understanding who will benefit most from this therapy and what potential synergistic treatment strategies should be further explored is challenging.

Hence, in this translational investigation our objective was to determine the association between HER2 expression, somatic mutations, and tumor immune microenvironment (TIME) factors in EOC. Additionally, we sought to determine how treatment with T-DXd impacted these same factors in ovarian cancer cell lines in an effort to identify potential combinatorial HER2-directed treatment strategies.

## Methods

### Clinical genomics patient specimens

**Figure 1:**
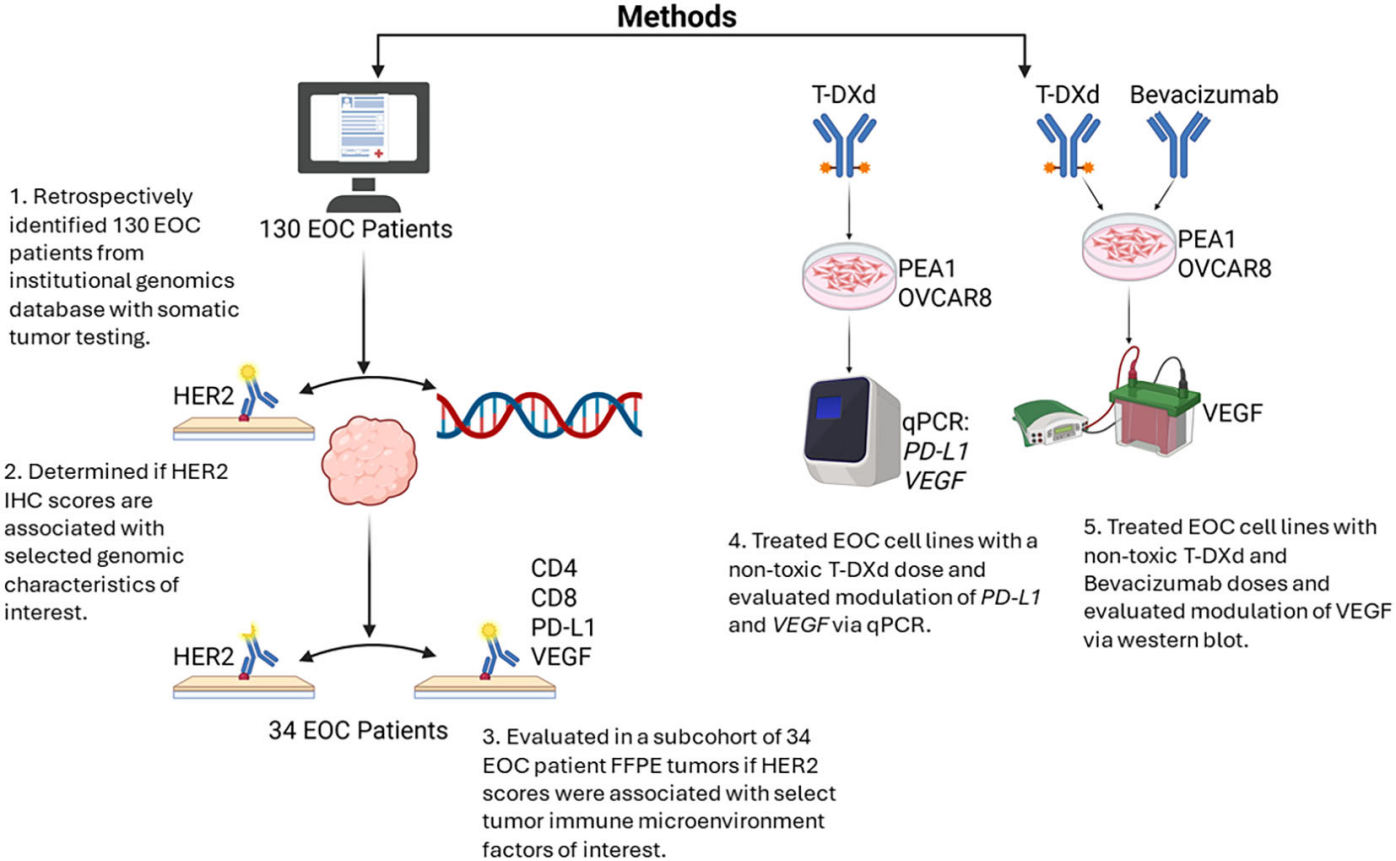
Experimental methods overview. Created in BioRender. James, N. (2026) https://BioRender.com/lq7vmzk.

An overview of experimental methods can be seen in **Figure 1**. To determine the association between HER2 pathologic scores and somatic tumor mutations, we obtained molecular profiling data from 130 patients with EOC from our institutional gynecologic cancer clinical genomics database. This database stores patient somatic tumor testing obtained from Caris Life Sciences since November 2022 in agreement with Women & Infants Institutional Review Board (IRB) approval which, in accordance with institutional provider consensus, is performed for all EOC patients as standard of care.

### Caris next-generation sequencing and immunohistochemistry

Detailed methods of Caris next-generation sequencing (NGS) and immunohistochemistry have been previously published (8). In brief, formalin-fixed, paraffin-embedded (FFPE) tumors from patients with EOC were sent to Caris for somatic tumor molecular profiling as standard of care at our institution and data were obtained retrospectively from our institutional gynecologic cancer clinical genomics database as described above. DNA-sequencing analysis was performed using an Illumina NovaSeq 6000 sequencer. Homologous recombination (HRD) status was defined from the HRD Genomic Scar Score which is calculated from the combination of total levels of genome-wide loss of heterozygosity (LOH) and large-scale chromosomal transitions (LST). LOH was calculated by splitting the 22 autosomal chromosomes into 552 segments and the LOH of single nucleotide polymorphisms (SNPs) within each segment is calculated. Caris data contains roughly 250,000 SNPs throughout the genome. SNP alleles with frequencies that are skewed towards 0% or 100% indicate LOH (heterozygous SNP alleles have a frequency of 50%). LSTs are reported when chromosomal breakages lead to chromosomal gains or losses of 10 Mb or greater. All Caris IHC assays are performed by a board-certified Caris pathologist; the Ventana HER2/neu IHC assay was used for HER2 staining and scoring was performed using the gastric ASCO/CAP criteria. HER2 high tumors were defined as having a pathologic score of 2+ or 3+, while HER2 low were defined as a pathologic score of 0 and 1 +. For PD-L1 (22c3) (pharmDx,DAKO) Caris IHC analysis, patients were considered positive if the combined positive score (CPS) was greater than or equal to 1. The threshold for FOLR1 (VENTANA FOLR1-2.1 RxDx, Ventana) positivity in Caris is defined as at least 75% of cells with 2+ staining intensity.

### Internal HER2 immunohistochemistry and scored patient specimens

HER2 IHC was performed by an expert gynecologic pathologist (KS) and independently reviewed and assigned a score of 0, 1+, 2+, 3+ using the gastric HER2 scoring criteria. A detailed overview of the HER2 gastric scoring system and full IHC methods can be seen in our group’s prior publication (8). For subsequent analyses HER2 scores were grouped as HER2 low (0 and 1+) and HER2 high (2+ or 3+).

A subset of thirty-four FFPE patient high grade serous ovarian tumors were obtained retrospectively under the auspice of Women & Infants IRB approval. All of these patients also had Caris testing available. These FFPE samples were previously stained for HER2 using gastric, breast, and endometrial scoring (8), and in the current study were used to determine the association between HER2 pathologic score and TIME factors via IHC.

### Fluorescent immunohistochemistry

Fluorescent IHC was employed to evaluate levels of CD8, CD4, VEGF, and PD-L1 in 34 high grade ovarian tumors as previously described (9, 10). FFPE tumors were baked for 2 hours at 65 °C and subsequently washed in SafeClear xylene substitute, 100% ethanol, 95% ethanol, 70% ethanol, deoxygenated water, and FTA Hemagglutination buffer for 10 minutes each on a shaker. Antigen retrieval was then performed using an antigen retrieval solution (1X; Vector Laboratories, H-3300) and heated at 95 °C for 20 minutes. Slides were blocked in 5% horse serum diluted in FTA Hemagglutination buffer and incubated overnight in primary antibody at 4 °C. Secondary antibody was then added for 1 hour in the dark at room temperature. Slide were cover-slipped with DAPI containing mounting medium. Primary and secondary antibodies along with respective dilutions were as follows: CD8 (Proteintech, 29896-1-AP, 1:50), PD-L1 (Proteintech, 66248-1-1g, 1:50), CD4 (Proteintech, 677886-1-1g, 1:50), VEGF (Abcam, ab1316, 1:50), Anti-Rabbit DyLight™488 (Vector Laboratories, DI-1488, 1:1,000), and Anti-Mouse DyLight™594 (Vector Laboratories, DI-2594, 1:1,000).

### Image analysis

To analyze PD-L1 and VEGF mean intensity, three random fields were selected based on DAPI staining using a 10x objective, including representative images. For CD4+ and CD8+ T cells, ten random fields were selected based on DAPI staining using a 40x objective including representative images. CD4+ and CD8+ T cells co-stained with DAPI positive cells were counted per field. All images were acquired with a Zeiss Axio Imager M1. Image J was employed for all image processing and analysis.

### Cell culture

The PEA1 cell line was obtained from Millipore Sigma and cultured in RPMI 1640 media supplemented with 2mM glutamine, 2 mM sodium Pyruvate, 10% fetal bovine serum (FBS), and 1% penicillin/streptomycin. OVCAR8 cells were obtained from American Type Culture Collection (ATCC) and were cultured in Dulbecco Modified Eagle Medium (DMEM) supplemented with 10% FBS and 1% penicillin/streptomycin. All cells were kept in a 37 °C/5% CO_2_ humidified chamber. Cells were treated with bevacizumab (Amgen), IgG-DXd (DAR 8) (MedChemExpress, cat#HY-164152A), and T-DXd (MedChemExpress, cat#HY-138298A) for 48 hours. All treatments used for downstream quantitative PCR and western blot analyses were first tested via a cell viability assay to ensure all treatments were non-toxic (**Supplementary Figure 1**).

### Western blot

Protein isolation, western blot, and associated analysis were performed as previously described (10). Briefly, protein was extracted from cells using Cell Lysis Buffer (Cell Signaling, 9803) containing 1mM of protease inhibitor cocktail (AbCam, ab6621). Concentrations of extracted proteins were determined by DC Protein Assay (Bio-Rad Laboratories, 5000116). Equal amounts of protein concentration were then boiled at 70 °C with Novex Sample Reducing agent (Life Technologies, NP009) and NuPAGE LDS sample buffer (ThermoFisher Scientific, NP0007). These samples were loaded into a 4-12% SurPAGE™ Bis-Tris Gel (GeneScript, M0056). Next, gels were transferred using a semi-dry method to methanol activated PVDF membrane through the employment of a Trans-Blot Turbo RTA Transfer Kit PVDF (Bio-Rad, 1704273) and the Bio-Rad Trans-Blot Turbo Transferring System (1.3A-25V) for 10 min. Membranes were subsequently blocked in 5% milk in phosphate-buffered saline with 0.05% Tween 20 (PBS-T) for 30 min at room temperature and primary antibodies diluted in 5% milk with PBS-T were incubated overnight at 4 °C. Secondary antibodies were then diluted in 5% milk in PBS-T for 1 hour at room temperature. Membranes were washed with PBS-T in between primary and secondary incubations and following secondary application. Clarity™ ECL substrate (Bio-Rad, 102030779,102030787) was applied to detect HRP-tagged secondary antibodies. The Bio-Rad ChemiDoc Imaging System was used to image all blots. All uncropped blots can be seen in **Supplementary Figure 2**. Antibodies and dilutions were as follows: GAPDH (Santa Cruz Biotechnology, 47724, 1:1000), VEGF (Proteintech, 81323-2-RR, 1:500), Anti-Rabbit Secondary (Cell Signaling, 7074S, 1:1000), and Anti-Mouse Secondary (Cell Signaling, 7076S, 1:1000).

### Quantitative PCR

RNA analysis and quantitative PCR(qPCR) were performed as previously described (9, 10). Briefly, RNA was isolated from cells using Trizol extraction and LiCl high salt precipitation. Total RNA (500ng) was reverse transcribed into cDNA via the iScript Synthesis kit (Bio-Rad, 1708890) following manufacturer’s protocol. qPCR was performed by loading 1µl of cDNA, 5µM of primers, and 10µl of SYBR Green (New England Biolabs, M3003E), and 5 µl of RNAse-free water to each well for Invitrogen primers. Validated Bio-Rad primers were used at a final 1X concentration with 10µl of SYBR Green and 8µl of RNAse-free water to each well. Samples were run on an ABI 7500 Fast-Real Time PCR system and all data was analyzed using the ΔΔCt method. Relative expression levels were normalized to 18s rRNA. Validated human primer pairs were purchased from BioRad (*VEGF* and *PD-L1*). 18s custom primers (Invitrogen) were as follows:18s rRNA-F-CCGCGGTTCTATTTTGTTGG and 18s rRNA-R-GGCGCTCCCTCTTAATCATG.

### Cell viability assays

Ovarian cancer cells were seeded in a 96-well plate (20,000 cells/well) and grown for 24 hours prior to treatment with IgG-DXd or T-DXd. After 48 hours, cells were exposed to 10µl per well of CellTiter 96^®^ Aqueous One Solution Cell proliferation MTS Assay (Promega, G3580), incubated for 1 hour at 37 °C/5% CO_2_, and finally absorbance read at 492nm to assess cell viability.

### Statistical analysis

GraphPad Prism was employed for all statistical analyses. Student t-tests were performed to determine mean differences between PD-L1, VEGF, CD8+ T cells and, CD4+ T cells according to HER2 low versus high pathologic scores. Fisher’s exact test was employed to determine how somatic mutational profiles differed according to HER2 score. Kaplan-Meier curves were used to determine how HER2 scores related to survival outcomes for patients in which follow-up information was available. All p-values reported were two-tailed and unadjusted, with p<0.05 representing a statistically significant result.

## Results

### Patient tumor genomic characteristics stratified by HER2 status

A total of 130 EOC patient tumors were included in the tumor molecular profile analysis.

Detailed patient characteristics are provided in **Table 1**.

**Table 1:** Genomics patient cohort clinical characteristics.

Age, median [range]	66 [21-89]
Platinum Free Interval (PFI), median [range]	14 [1-102]
Overall Survival (OS), median [range]	26.5 [6-176]
Histology, N (%)	High-Grade Serous, 98 (75.4%)
	Low-Grade Serous, 12 (9.2%)
	Carcinosarcoma, 3 (2.3%)
	Endometrioid, 7 (5.4%)
	Mucinous, 4 (3.1%)
	Clear Cell, 4 (3.1%)
	Mesonephric, 2 (1.5%)
Stage, N (%)	I, 24 (18.5%)
	II, 15 (11.5%)
	III, 67 (51.5%)
	IV, 17 (13.1%)
	Advanced NOS, 7 (5.4%)
HER2 Score, N (%)	0-1, 101 (78%)
	2-3, 29 (22%)

In this cohort, 22% were HER2 high (n=29) and 78% (n=101) were HER2 low (**Table 1**). There were no significant differences observed in platinum-free interval (PFI) or overall survival (OS) between patients with a HER2 high versus low score (**Supplementary Figure 3**) Non-significant differences (p=0.77) were observed in HRD positivity with 17% of HER2 high patients HRD positive and 69% HRD negative. This proportion was similar in HER2 low patients, as 16% and 76% were defined as HRD positive and negative, respectively. HER2 status was not associated with either *CCNE1* amplification or pathogenic *ARID1A* mutations; 7% of HER2 high and low tumors were positive for *CCNE1* amplification and 7% and 9% of HER2 high and low tumors, respectively, harbored an *ARID1A* pathogenic mutation. Interestingly, although non-significant (p=0.44), it was observed that a higher proportion of HER2 high tumors were PD-L1 positive compared to HER2 low tumors (57% vs 48%). This phenomenon was even more striking when the PD-L1 CPS cut-off was increased to 5, in which 33% of HER2 high patients were PD-L1 positive compared to only 18% of HER2 low patients (p=0.14) (**Supplementary Table 1**), suggesting that a higher proportion of EOC tumors were positive for both HER2 and PD-L1. These data provided the basis for the translational studies described below, in which the relationship between HER2 status, HER2-directed therapies, and PD-L1 was further investigated. Lastly, we determined that only 23% of HER2 high tumors were also FOLR1 positive, compared to 41% of HER2 low tumors (p=0.11), indicating that HER2 low tumors were more likely to overexpress FOLR1. A complete list of values can be seen in **Table 2**.

**Table 2:** EOC patient cohort of selected ovarian genomic characteristics stratified by HER2 pathologic score.

Ovarian genomic characteristic	HER2 high (n=29)	HER2 low (n=101)	p-value
HRD			
Indeterminate	4 (14%)	9 (9%)	0.77
Negative	20 (69%)	76 (76%)	
Positive	5 (17%)	16 (16%)	
CCNE1			
Non-amplified	21 (72%)	76 (76%)	>0.99
Amplified	2 (7%)	7 (7%)	
Intermediate	6 (21%)	18 (18%)	
ARID1A			
Wild type	27 (93%)	92 (2%)	>0.99
Pathogenic Variant	2 (7%)	9 (9%)	
PD-L1			
Negative	9 (43%)	46 (52%)	0.48
Positive	12 (57%)	42 (48%)	
FOLR1			
Negative	20 (77%)	57 (59%)	0.11
Positive	6 (23%)	40 (41%)	

Next, we sought to determine the association between HER2 status and genomic outcomes in a sub-analysis of patients with high grade serous ovarian cancer (HGSOC, n=98) (**Table 2**), of which 21% (n=21) and 79% (n=77) were HER2 low and high, respectively. These percentages were similar to those of the entire EOC cohort. Also consistent with the results from the EOC cohort, non-significant (p=0.53) differences were observed when HRD positivity was stratified by HER2 status. Similar non-significant differences were also observed for *CCNE1* amplification (p=0.33) status and *ARID1A* mutations (p=0.38) stratified by HER2 status.

Although less striking compared to the EOC cohort, patients that were HER2 high similarly had a slightly higher percentage of PD-L1 positivity compared to HER2 low patients (63% versus 57%, p=0.78). This trend also held true when PD-L1 positivity was increased to a CPS score of 5, in which 31% of HER2 high versus 23% of HER2 low patients were positive (p=0.53) (**Supplementary Table 2**). Overall, this observed decrease in PD-L1 differences would be expected in a HGSOC cohort as it has been well documented that non-serous EOC tumors, such as endometroid and clear cell, are more likely to be PD-L1 positive (11). Finally, similar differences in FOLR1 positivity were observed in our HGSOC cohort as 30% versus 49% of tumors were FOLR1 positive in HER2 high and low tumors, respectively (p=0.14). A complete list of values can be seen in **Table 3**.

**Table 3:** HGSOC patient cohort of selected genomic characteristics stratified by HER2 pathologic score.

Ovarian genomic characteristic	HER2 high (n=21)	HER2 low (n=77)	p-value
HRD			
Indeterminate	4 (14%)	8 (10%)	0.53
Negative	12 (57%)	54 (70%)	
Positive	5 (24%)	15 (30%)	
CCNE1			
Non-amplified	16 (76%)	52 (68%)	0.33
Amplified	0 (0%)	7 (9%)	
Intermediate	5 (24%)	18 (23%)	
ARID1A			
Wild type	20 (95%)	76 (99%)	0.38
Pathogenic Variant	1 (5%)	1 (1%)	
PD-L1			
Negative	6 (37%)	28 (43%)	0.78
Positive	10 (63%)	36 (57%)	
FOLR1			
Negative	14 (70%)	38 (51%)	0.14
Positive	6 (30%)	37 (49%)	

### Intratumoral expression of TIME factors stratified by HER2 status

**Figure 2:**
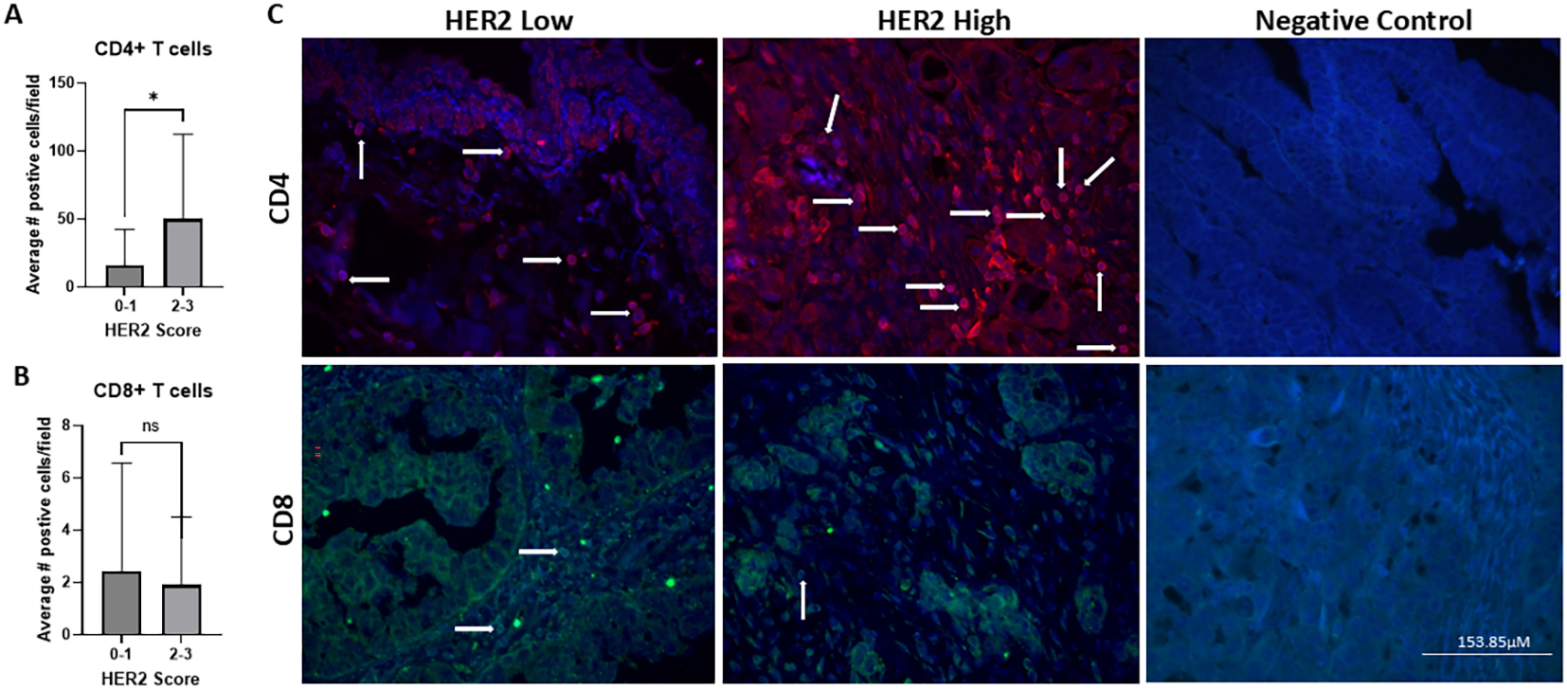
CD4+ and CD8+ T cell expression stratified by HER2 pathologic score in EOC tumors. Fluorescent immunohistochemistry analysis of **(A)** CD4+ and **(B)** CD8+ T cells in HER2 low (0,1; n=23) and HER2 high (2,3; n=11) EOC tumors, denoted by the number of positive cells per field. **(C)** Representative images with arrows depicting examples of positive CD4+ or CD8+ T cells. *ns, non-significant *p<0.05, as indicated.

A subset of EOC tumors (n=34) were utilized for fluorescent IHC analysis of pertinent TIME factors stratified by HER2 high (n=23) versus low (n=11) pathologic scores. Detailed clinical outcomes for this subset of patients can be seen in **Table 4**. Similar to the genomic patient cohort, there were no significant differences in PFI, or OS upon stratification of patients with a HER2 high versus low score (**Supplementary Figure 4**) Interestingly, it was found that patients with a HER2 high score had significantly (p=0.029) higher mean levels of CD4+ T cell cells (50.27 average number of T cells per field) compared to HER2 low tumors (15.91 average number of T cells per field) (**Figure 2a**). Non-significant (p=0.127) differences in CD8+ T cells were detected between HER2 high versus low tumors (**Figure 2b**). Representative images of CD4+ and CD8+ T cells intratumoral staining can be seen in **Figure 2c**.

**Table 4:** Fluorescent immunohistochemistry patient cohort clinical characteristics.

Age, median [range]	68 [44-89]
Platinum Free Interval (PFI), median [range]	14 [1-35]
Overall Survival (OS), median [range]	32 [10-48]
Histology, N (%)	High-Grade Serous, 33 (97%)
	Carcinosarcoma 1 (3%)
Stage, N (%)	I, 7 (20%)
	II, 4 (12%)
	III, 16 (47%)
	IV, 4 (12%)
	Advanced NOS, 3 (9%)
HER2 Score, N (%)	0-1, 23 (68%)
	2-3, 11 (32%)

**Figure 3:**
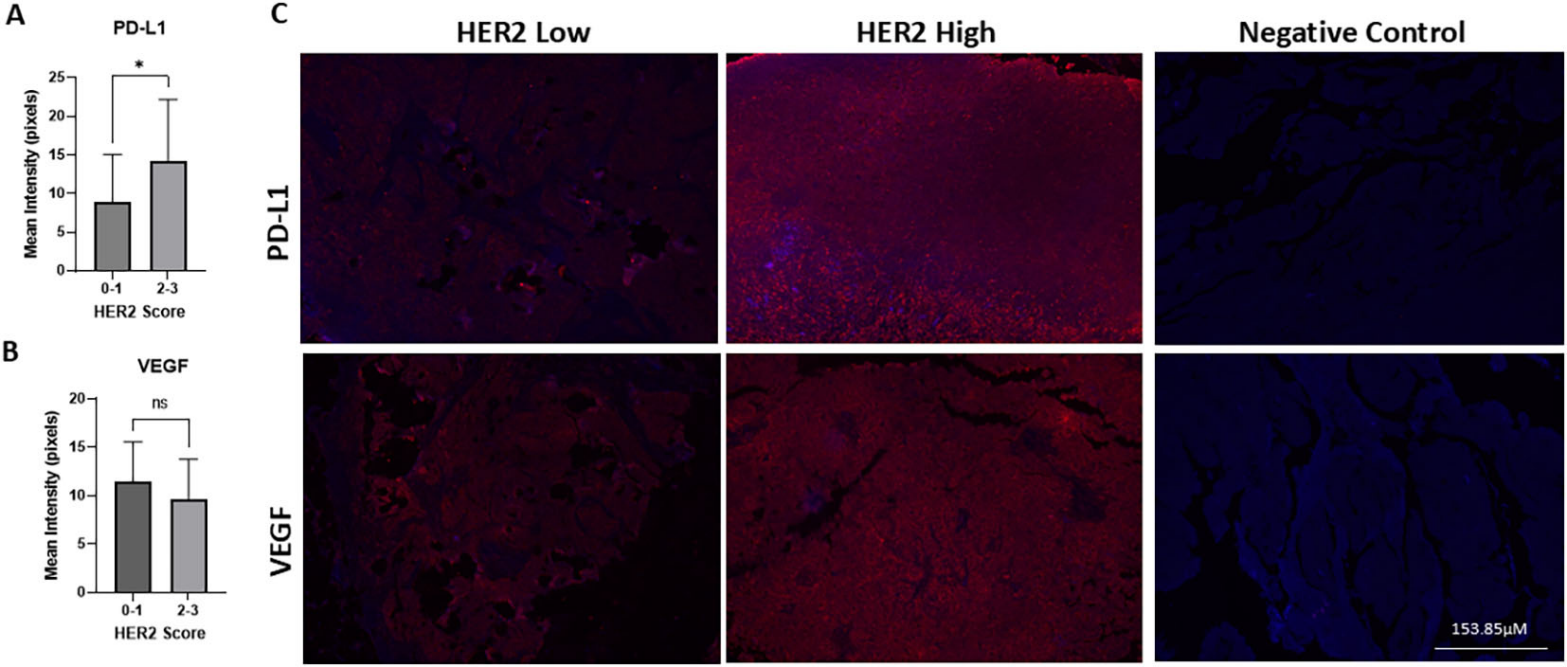
PD-L1 and VEGF intratumoral expression stratified by HER2 pathologic score in EOC tumors. Fluorescent immunohistochemistry analysis of **(A)** PD-L1 and **(B)** VEGF HER2 low (0,1; n=23) and HER2 high (2,3; n=11) EOC tumors, denoted by mean intensity(pixels). **(C)** Representative images of PD-L1 and VEGF intratumoral staining. *ns, non-significant *p<0.05, as indicated.

We then sought to evaluate intratumoral expression of clinically relevant microenvironment factors, PD-L1 and VEGF, in EOC to determine if levels were associated with patient HER2 scores. It was determined that significantly (p=0.0379) higher mean intensity levels of PD-L1 were found in patients with HER2 high tumors (14.29 pixels) versus patient tumors with a HER2 low score (8.93 pixels, **Figure 3a**). However, upon stratifying VEGF intratumoral expression by HER2 score, no significant differences were identified (p=0.246, **Figure 3b**). Representative images of PD-L1 and VEGF intratumoral staining can be seen in **Figure 3c**. Despite this limited cohort size, these results suggest that HER2 pathologic score is associated with higher PD-L1 expression, which was consistent with patient somatic tumor data.

### T-DXd modulates PD-L1 and VEGF expression

**Figure 4:**
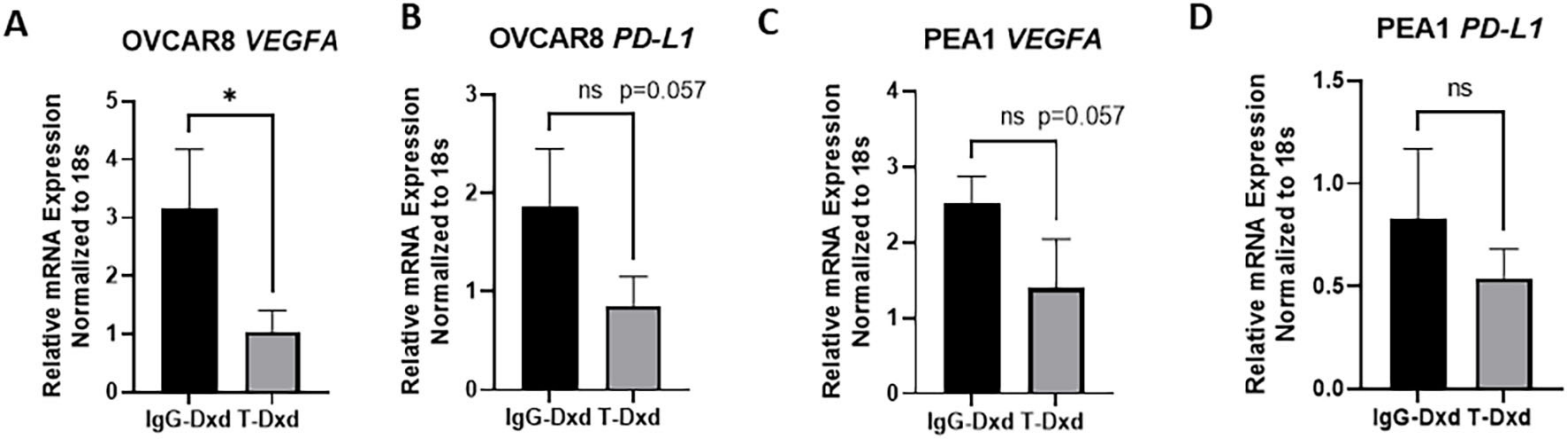
T-DXd modulates PD-L1 expression. Quantitative PCR analysis of **(A)** *VEGFA* and **(B)** *PD-L1* in OVCAR8 cells treated with 0.8ug/ml of IgG-DXd or T-DXd for 48-hours. Quantitative PCR analysis of **(C)** *VEGFA* and **(D)** *PD-L1* in PEA1 cells treated with 0.2ug/ml of IgG-DXd or T-DXd for 48-hours. *ns, non-significant *p<0.05, as indicated.

**Figure 5:**
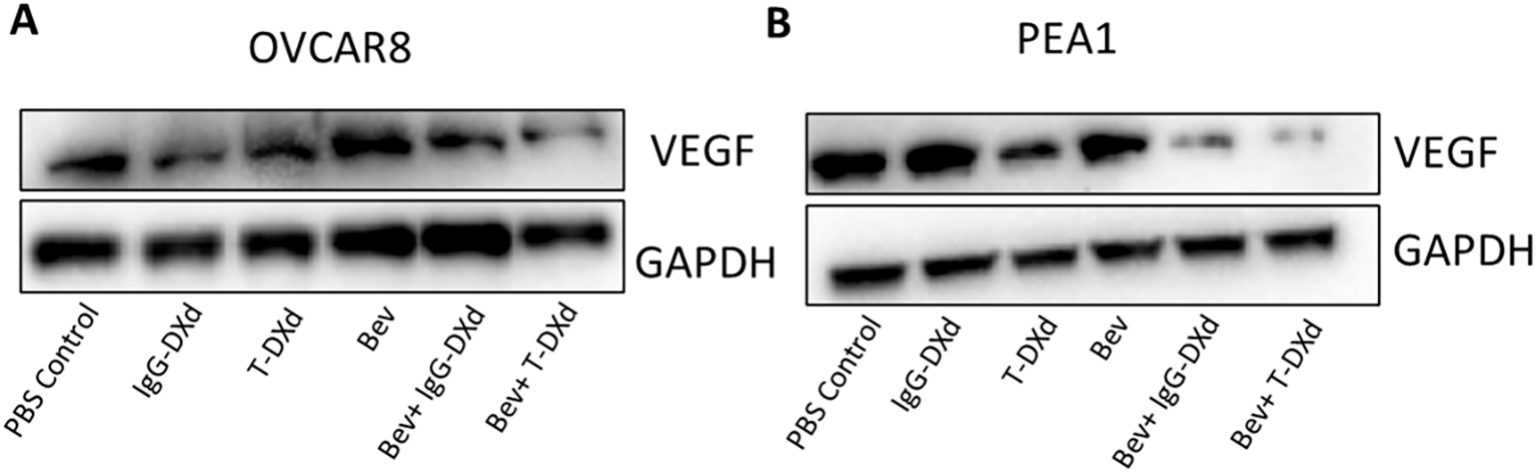
T-DXd modulates VEGF expression. Western blot analysis of VEGF and corresponding GAPDH loading control in **(A)** OVCAR8 and **(B)** PEA1 cells treated with 5ug/ml of bevacizumab and 0.8ug/ml or 0.2ug/ml of IgG-DXd/T-DXd, respectively.

**Figure 6:**
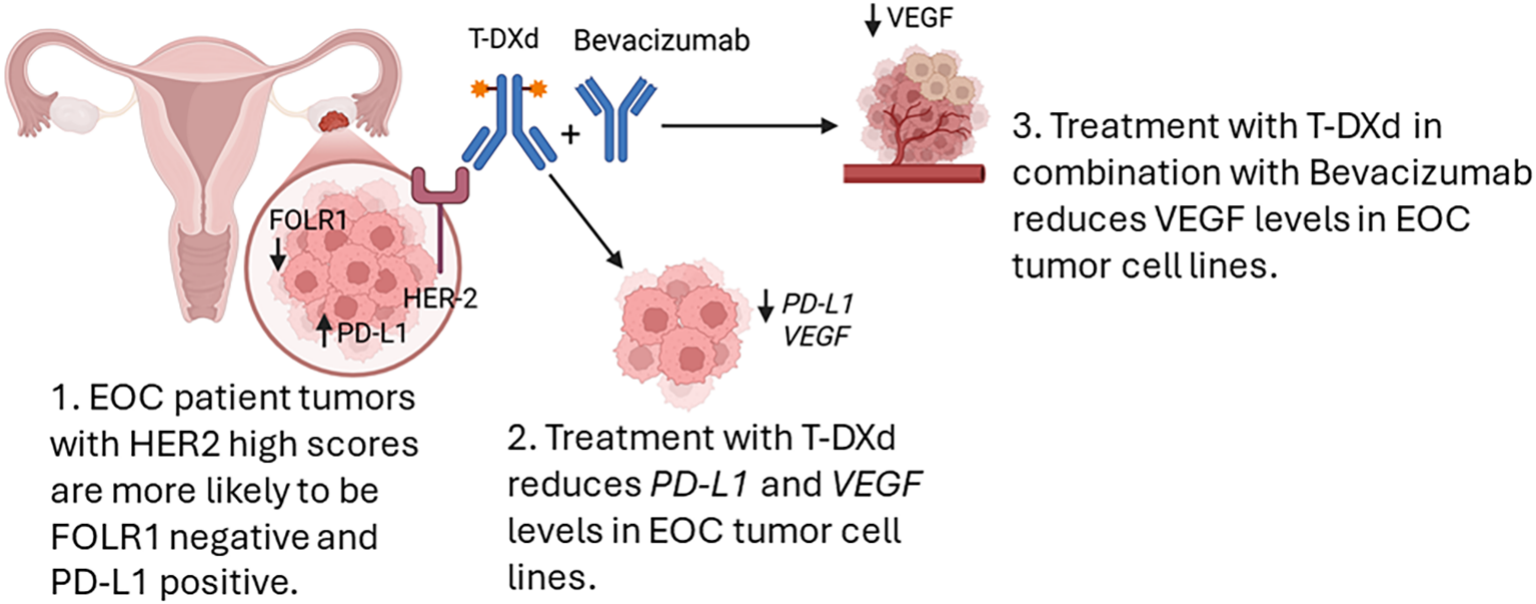
Summary of key findings. Created in BioRender. James, N. (2026) https://BioRender.com/pjj0yrn.

Next, we sought to determine if T-DXd modulated PD-L1 and VEGF in EOC cell lines. PEA1, OVCAR8, and PEA2 cells were tested for basal levels of HER2 expression, which revealed highest levels in PEA1 cells with second highest expression levels in OVCAR8 cells (**Supplementary Figure 5**). Hence, we proceeded with these cell lines for subsequent downstream quantitative PCR analyses. In OVCAR8 cells, treatment with T-DXd led to a significant (p=0.039) 3.02-fold reduction in *VEGFA* expression (**Figure 4a**) and a substantial (p=0.057) 2.18-fold reduction in *PD-L1* expression (**Figure 4b**) compared to IgG-DXd control. Similar trends were observed in PEA1 cells which revealed substantial but non-significant 1.79-fold and 1.53-fold reductions in *VEGFA* and *PD-L1* expression, respectively (**Figures 4c, d**). Western blot analysis was then performed on OVCAR8 (**Figure 5a**) and PEA1 (**Figure 5b**) cells treated with IgG-DXd and T-DXd alone and in combination with bevacizumab, which revealed decreased VEGF expression in both combinatorial IgG-DXd and T-DXd, particularly in the PEA1 cells, suggesting that the DXd payload may synergize with standard of care maintenance EOC therapy bevacizumab to reduce VEGF expression. Therefore, T-DXd and bevacizumab may be an effective combinatorial treatment approach in the maintenance or recurrent setting. A graphical summary of all findings can be seen in **Figure 6**.

## Discussion

Although T-DXd is increasingly being used to treat chemoresistant HER2-expressing ovarian cancer following the pan tumor drug approval, there have been very few translational and preclinical mechanistic studies designed to understand how HER2 status and treatment with T-DXd impact EOC tumor cells. In the first part of this study, we aimed to determine what clinically relevant genomic characteristics and TIME factors were associated with HER2 expression in EOC to potentially identify patients that may benefit most from single agent or combinatorial HER2-directed therapy and to provide the foundation for our subsequent translational endpoints. While the definition of HER2 status varies across clinical trials and other studies, for this current investigation we chose to define HER2 2+ and 3+ as HER2-high based on the DESTINY-PanTumor02 and STATICE trials. Hence, ISH results were not included, as they were not performed for all patients and importantly, in these recent trials stratification emphasizes HER2 IHC score regardless of ISH results (5, 12).

To the best of our knowledge, there have only been two previous studies that have evaluated HER2 status according to *BRCA1/2* mutational or HRD status. The first was a study by Kim et al. which determined that HER 3+ tumors were exclusively defined as *BRCA* wildtype (13), while a second study found that HER2 2+ and 3+ tumors were more likely to be HRD positive (14). This current investigation suggested non-significant differences in HRD status based on HER2 status, adding to the discordance in the literature of HER2’s association with HRD status in EOC. Despite our relatively small sample size being a study limitation, a considerable strength was the uniformity of our data as all HER2 scores and ovarian genomic characteristic data were obtained from the same tumor and timepoint, unlike prior published analyzes (13, 14).

One of the most prominent trends from this study was that HER2 high expression was associated with reduced FOLR1 positivity, although not statistically significant. This finding was corroborated by Park et al. who similarly found that concurrent high FOLR1 and HER2 expression was rare (14). Overall, as mirvetuximab soravtansine (MIRV) is an approved ADC for FOLR1 high platinum resistant EOC tumors based on efficacy demonstrated by the MIRASOL trial (15), it appears as though distinct EOC patient populations would initially benefit from MIRV versus T-DXd therapy. ADC’s are beings investigated in ongoing trials to examine efficacy of these agents in earlier lines of therapy, in the neoadjuvant and platinum sensitive settings; future directions include understanding the antibody sequencing implications of MIRV and T-DXd therapy to determine if either modulates levels of HER2 or FOLR1.

This analysis also revealed that HER2 high EOC tumors were more likely to be PD-L1 positive. Building on this clinical finding, fluorescent IHC analysis in a smaller cohort corroborated these results. Furthermore, we found that treatment with T-DXd treatment reduced *PD-L1* expression in two HGSOC cell lines. Consistent with our data, a prior study in ovarian cancer found that subsets of HGSOCs had positive PD-L1 intratumoral expression coupled with HER2 amplification (16). Conversely, Kim et al. reported that HER2 3+ EOC tumors were also PD-L1 low, however similar to HRD status, this analysis was not performed on uniform samples or with standardized HER2 staining methods (13). Overall, our results provide preclinical support for consideration of combinatorial PD-1 based immunotherapy and T-DXd treatment for patients with HER2 high EOC. However, the fact that we observed reduced levels of *PD-L1* expression following T-DXd, may biologically contradict sensitivity to PD-1-based immunotherapy if intratumoral PD-L1 levels are reduced. Hence, the molecular mechanism of targeting T-DXd in combination with PD-1-based immunotherapy warrants further investigation. Furthermore, as we examined tumor-intrinsic modulation of *PD-L1* following T-DXd treatment, this experiment should be repeated using *in vivo* models that recapitulate the ovarian TIME. Overall, the association between HER2 and PD-L1 is clinically timely given the recent FDA approval of pembrolizumab for platinum-resistant ovarian cancer in combination with paclitaxel (with or without bevacizumab) for patients with PD-L1 expressing tumors (CPS≥1) following results from the KEYNOTE-B96 trial (17). A recently published pre-clinical study also found that inetetamab, a humanized HER2 monoclonal antibody with a Fc engineered region that enhances antibody-dependent cell-mediated toxicity, synergizes with atezolizumab in murine ovarian cancer models and creates durable anti-tumor immune responses by elevating CD103+CD8+ infiltration levels. In addition, this study demonstrated a significant correlation between intratumoral HER2 and PD-L1, CD4+, and CD8+ expression in EOC patient tissue samples (18). Interestingly, our IHC analysis revealed that HER2 pathologic scores were correlated with CD4+ lymphocytes and PD-L1 expression, but not CD8+ T cells. However, our analysis had a smaller sample size. Taken together, these studies support our conclusion that dual HER2 and PD-1/PD-L1 targeting may represent a novel therapeutic combination in EOC with a strong preclinical foundation. While clinical trials are investigating dual T-DXd and anti-PD-1/PD-L1 targeting in gastric (NCT0437996), metastatic triple-negative breast cancer (NCT03742102), and non-small cell lung cancer (NCT06899126), this combination has yet to be tested clinically in EOC.

Finally, despite the fact that intratumoral VEGF levels were not found to be significantly different when stratified by HER2 expression, we found that *VEGFA* was meaningfully downregulated by T-DXd treatment in two HGSOC cell lines with differing levels of HER2 expression. Furthermore, we found that both IgG-DXd and T-DXd synergized with bevacizumab to reduce VEGF protein expression, indicating that T-DXd’s payload reduces VEGF expression. Thus, a HER2 specific mediated effect may be limited. Interestingly, a preclinical study by Li et al. found that dual targeting with the VEGFR inhibitor cediranib maleate and HER2-ADC RC48 promoted tumor cell death via the PI3K-AKT pathway (19). Taken together, these results suggest that further preclinical mechanistic studies should be performed to investigate the potential synergism between T-DXd and bevacizumab. Interestingly, in 2025 a clinical trial was initiated to investigate T-DXd and bevacizumab versus bevacizumab monotherapy for first-line maintenance in HER2-expression ovarian cancer (DESTINY-Ovarian1, NCT06819007). Results from this trial and corresponding translational endpoint studies will be highly anticipated.

### Study limitations

The primary limitation in this current study was the small sample size of HER2 high scores both in our clinical genomic and IHC patient cohort. This is a persistent challenge as approximately 30-34% of EOC patients are considered HER2 high (8). In particular, in our IHC cohort we were limited by the inclusion of only 11 HER2 high EOC patient samples. This small sample size potentially could have contributed to the fact that we did not observe statistically significant differences in the number of CD8+ T cells in our HER2 high versus low EOC patient tumors. Similarly, if we had an expanded clinical genomic patient cohort, we may have observed statistically significant differences in FOLR1 and PD-L1 expression when stratified by HER2 status. Hence, future directions stemming from this project include the expansion of this analysis to reevaluate genomic characteristics and TIME factors of interest, as well as determine levels of other commonly investigated ADC targets in gynecologic malignancies such as TROP2, B7-H4 and Claudin-6.

## Conclusion

This study identified key genomic characteristics and TIME factors associated with high HER2 expression in EOC tumors. Our analysis demonstrated that the majority of HER2 high tumors were FOLR1 negative, but that HER2 expression was associated with a higher level of PD-L1 positivity. Finally, we showed that T-DXd treatment reduced levels of *PD-L1* and *VEGF* expression. Taken together, these results suggest that patients with HER2 high tumors may potentially also be candidates for anti-PD-1/PD-L1 therapy and that T-DXd may synergize with bevacizumab to reduce VEGF expression in EOC. Ultimatley, this study represents a key first step in identifying characteristics of HER2 high tumors in EOC in order to provide personalized combinatorial treatment approaches for patients. As more ADCs are in the process of being clinically investigated for the treatment of ovarian and gynecologic malignancies, performing companion translational and mechanistic endpoint studies will be of critical importance to the improvement of patient outcomes.

## Data Availability

The original contributions presented in the study are included in the article/**Supplementary Material**. Further inquiries can be directed to the corresponding author.

## Supplementary material

Supplementary Figure 1 Cell viability analysis of IgG-DXd and T-DXd in PEA1 and OVCAR8 cells. Cell viability analysis of **(A)** PEA1 and **(B)** OVCAR8 cells, treated with 0.2ug/ml and 0.8ug/ml of IgG-DXd/T-DXd, in PEA1 and OVCAR8 cells, respectively.

Supplementary Figure 2 Uncropped western blot images.

Supplementary Figure 3 Genomic patient cohort survival outcomes stratified by HER2 score. Kaplan-Meier survival curve analysis of HER2 score association with **(A)** platinum-free interval, stratified by HER2 low (n=24) and HER2 high (n=84) scores and **(B)** overall survival, stratified by HER2 low (n=81) and HER2 high (n=24) scores.

Supplementary Figure 4 Immunohistochemistry patient cohort survival outcomes stratified by HER2 score. Kaplan-Meier survival curve analysis of HER2 score association with **(A)** platinum-free interval, stratified by HER2 low (n=9) and HER2 high (n=20) scores and **(B)** overall survival, stratified by HER2 low (n=19) and HER2 high (n=8) scores.

Supplementary Figure 5 Basal levels of HER2 expression in HGSOC cell lines.
