# Supplementary figures and images for: Association between HER2 expression, genomic characteristics, and tumor immune microenvironment dynamics in epithelial ovarian cancer
Source: Front Oncol. 2026 Jul 20;16:1867768. doi: 10.3389/fonc.2026.1867768 (PMC13429427; doi:10.3389/fonc.2026.1867768)

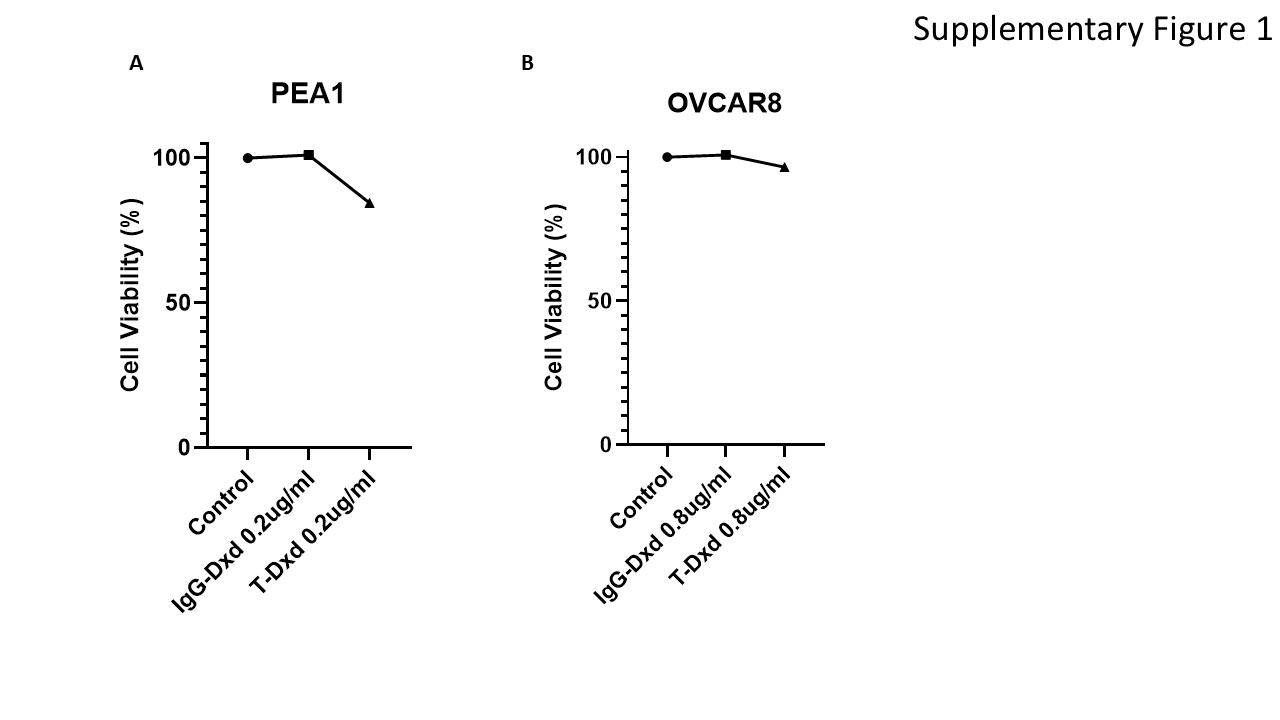

Supplement: Supplementary Figure 1 — Cell viability analysis of IgG-DXd and T-DXd in PEA1 and OVCAR8 cells. Cell viability analysis of (A) PEA1 and (B) OVCAR8 cells, treated with 0.2ug/ml and 0.8ug/ml of IgG-DXd/T-DXd, in PEA1 and OVCAR8 cells, respectively. [file Image1.tif]

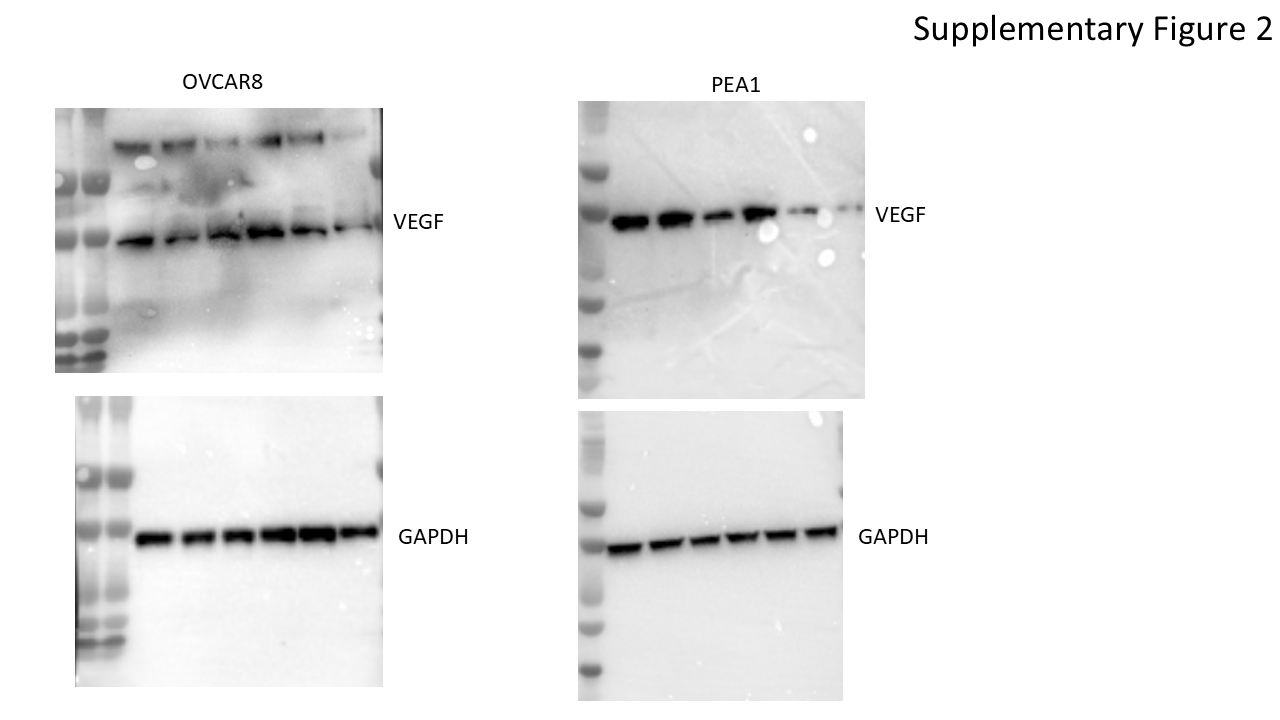

Supplement: Supplementary Figure 2 — Uncropped western blot images. [file Image2.tif]

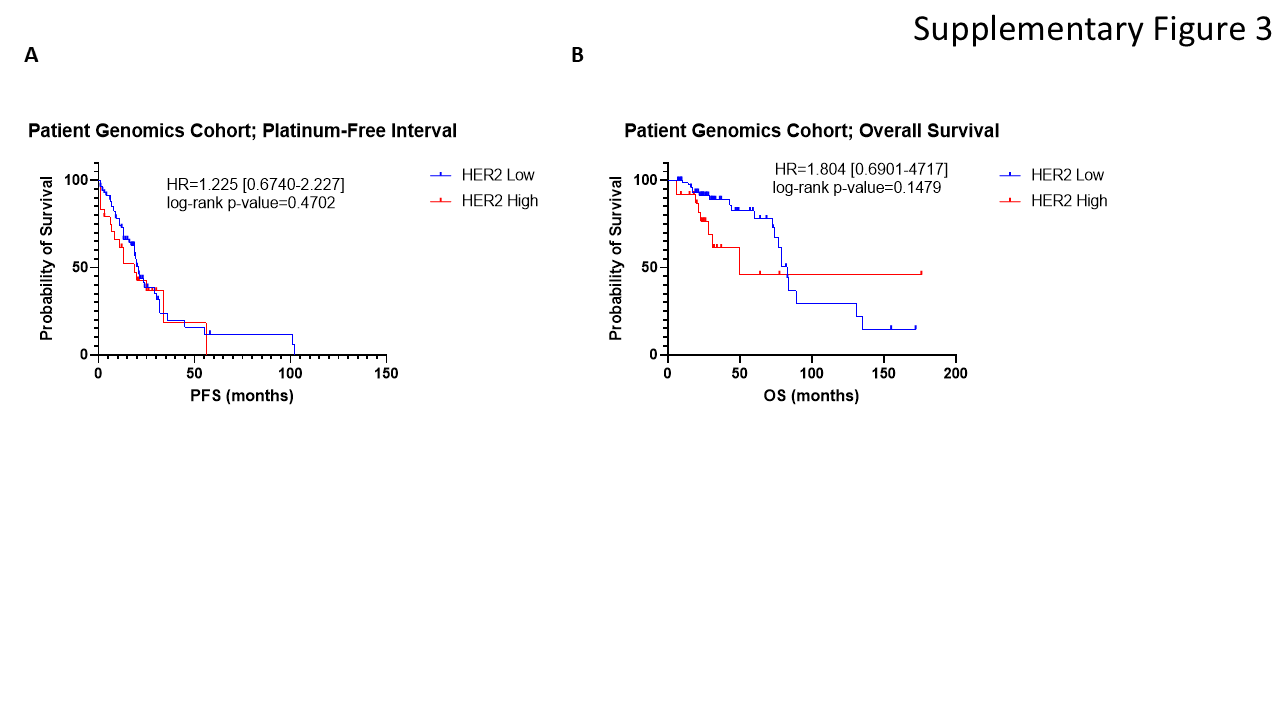

Supplement: Supplementary Figure 3 — Genomic patient cohort survival outcomes stratified by HER2 score. Kaplan-Meier survival curve analysis of HER2 score association with (A) platinum-free interval, stratified by HER2 low (n=24) and HER2 high (n=84) scores and (B) overall survival, stratified by HER2 low (n=81) and HER2 high (n=24) scores. [file Image3.tif]

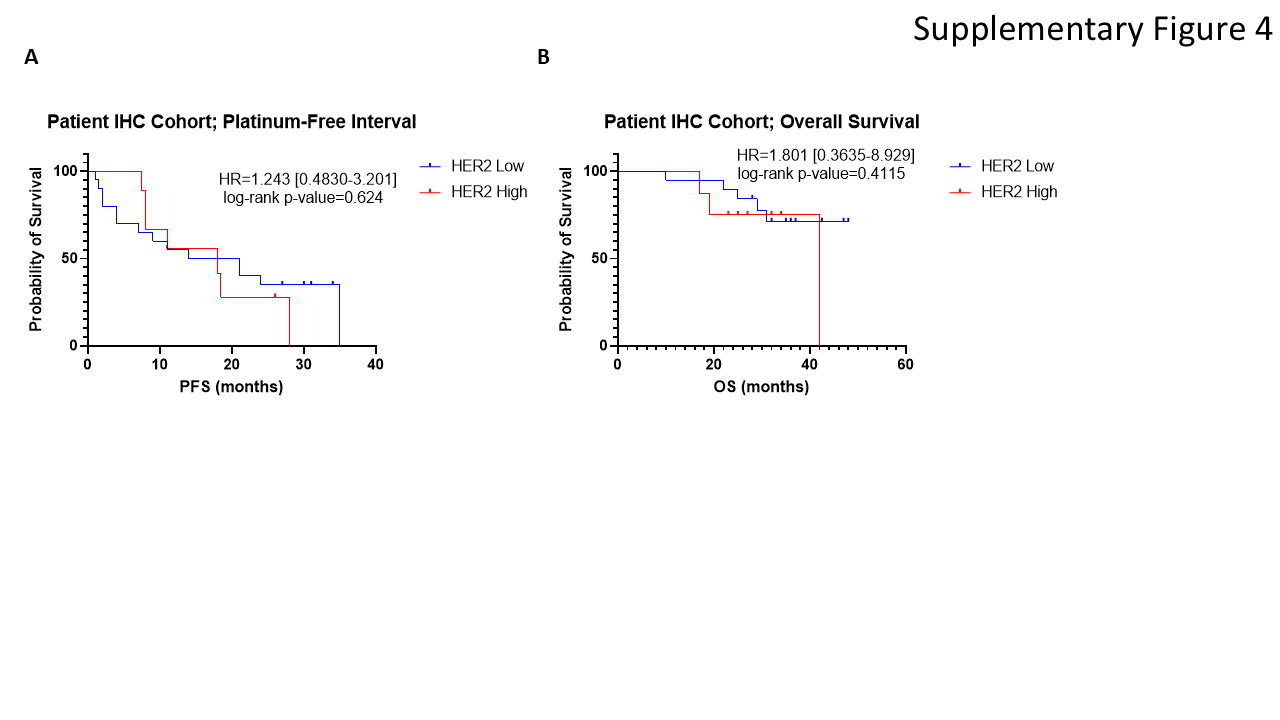

Supplement: Supplementary Figure 4 — Immunohistochemistry patient cohort survival outcomes stratified by HER2 score. Kaplan-Meier survival curve analysis of HER2 score association with (A) platinum-free interval, stratified by HER2 low (n=9) and HER2 high (n=20) scores and (B) overall survival, stratified by HER2 low (n=19) and HER2 high (n=8) scores. [file Image4.tif]

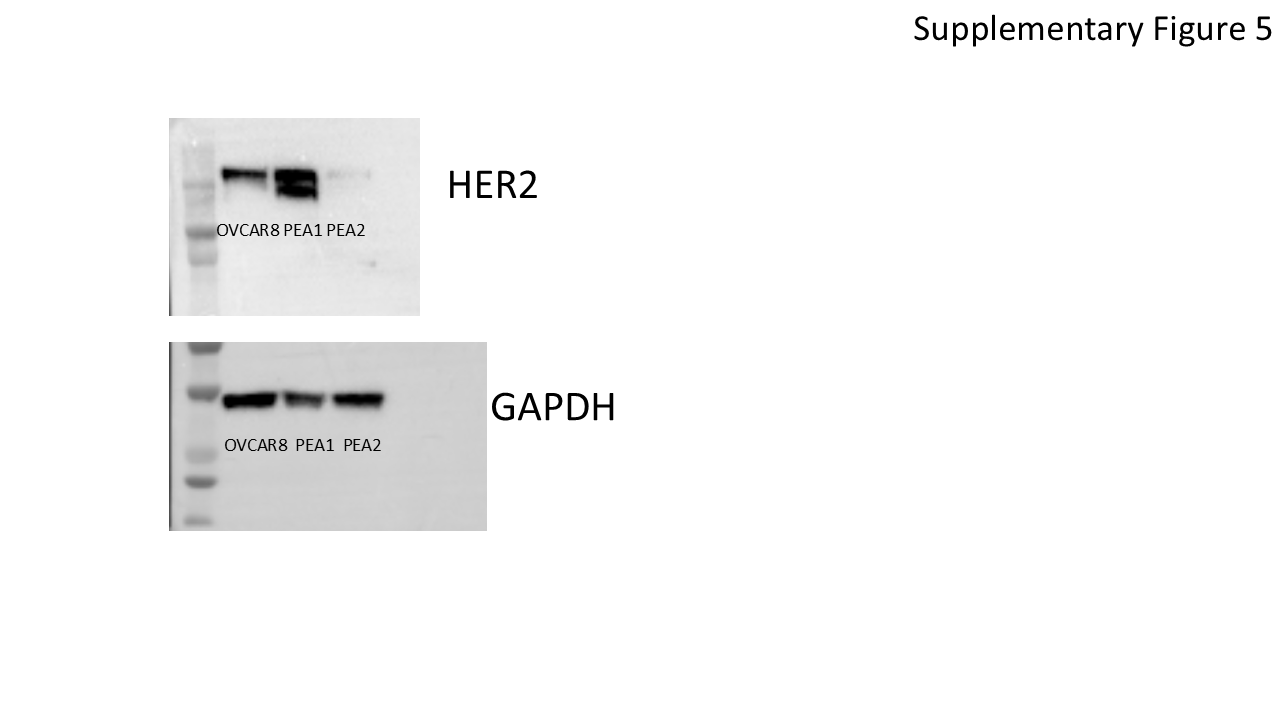

Supplement: Supplementary Figure 5 — Basal levels of HER2 expression in HGSOC cell lines. [file Image5.tif]
