# Supplementary material for: Association between HER2 expression, genomic characteristics, and tumor immune microenvironment dynamics in epithelial ovarian cancer
Source: Front Oncol. 2026 Jul 20;16:1867768. doi: 10.3389/fonc.2026.1867768 (PMC13429427; doi:10.3389/fonc.2026.1867768)
Supplement: Supplementary file 7 [file Table2.docx]

**Supplementary Table 2. HGSOC patient PD-L1 combined positive score (CPS) score ≥5 stratified by HER2 pathologic score.**

| **Ovarian Genomic Characteristic** | **HER2 High (n=21)** | **HER2 Low (n=77)** | **p-value** |
| --- | --- | --- | --- |
| PD-L1 CPS Score ≥ 5 |  |  | 0.53 |
| Negative | 11 (69%) | 49 (77%) |  |
| Positive | 5 (31%) | 15 (23%) |  |
